# Interference with Bacterial Conjugation and Natural Alternatives to Antibiotics: Bridging a Gap

**DOI:** 10.3390/antibiotics12071127

**Published:** 2023-06-29

**Authors:** Micaela Guidotti-Takeuchi, Roberta Torres de Melo, Lígia Nunes de Morais Ribeiro, Carolyne Ferreira Dumont, Rosanne Aparecida Capanema Ribeiro, Bárbara de Araújo Brum, Tanaje Luiz Izidio Ferreira de Amorim Junior, Daise Aparecida Rossi

**Affiliations:** 1 Laboratory of Molecular Epidemiology, Federal University of Uberlândia, Uberlândia 38402-018, MG, Brazil; carolyne.dumont@ufu.br (C.F.D.); rosanneapda@ufu.br (R.A.C.R.); barbara.brum@ufu.br (B.d.A.B.); tanaje@ufu.br (T.L.I.F.d.A.J.); daise.rossi@ufu.br (D.A.R.); 2 Institute of Biotechnology, Federal University of Uberlândia, Uberlândia 38405-320, MG, Brazil; ligia.ribeiro@ufu.br

**Keywords:** bacterial conjugation, *bla_TEM_*, chicken juice, nanostructured lipid carriers, whey

## Abstract

Horizontal gene transfer (HGT) in food matrices has been investigated under conditions that favor gene exchange. However, the major challenge lies in determining the specific conditions pertaining to the adapted microbial pairs associated with the food matrix. HGT is primarily responsible for enhancing the microbial repertoire for the evolution and spread of antimicrobial resistance and is a major target for controlling pathogens of public health concern in food ecosystems. In this study, we investigated *Salmonella* Heidelberg (SH) and *Escherichia coli* (EC) regarding gene exchange under conditions mimicking the industrial environment, with the coproducts whey (SL) and chicken juice (CJ). The *S.* Heidelberg strain was characterized by antibiotic susceptibility standards and PCR to detect the *bla*_TEM_ gene. A concentration of 0.39 mg/mL was determined to evaluate the anti-conjugation activity of nanostructured lipid nanocarriers (NLCs) of essential oils to mitigate β-lactam resistance gene transfer. The results showed that the addition of these coproducts promoted an increase of more than 3.5 (whey) and 2.5 (chicken juice) orders of magnitude in the conjugation process (*p* < 0.01), and NLCs of sage essential oil significantly reduced the conjugation frequency (CF) by 74.90, 90.6, and 124.4 times when compared to the transfers in the absence of coproducts and the presence of SL and CJ, respectively. For NLCs from olibanum essential oil, the decrease was 4.46-fold for conjugations without inhibitors and 3.12- and 11.3-fold in the presence of SL and CJ. NLCs associated with sage and olibanum essential oils effectively control the transfer of antibiotic resistance genes and are a promising alternative for use at industrial levels.

## 1. Introduction

Antimicrobial resistance (AMR) is one of the main global threats today, and recent reports estimate that it directly caused 1.27 million deaths in 2019 [1]. In Brazil, the National Health Surveillance Agency, through the Evaluation of National Indicators of Health Care-Related Infections (HAIs) and Microbial Resistance (MR), in the year 2021, estimated that *Escherichia coli* resistance to different classes of beta-lactams can reach rates of 47.5% to cephalosporins, 16.5% to carbapenems in hospital settings [2], and 74.28% and 62.85% to ampicillin and amoxicillin, respectively, in samples from humans and animals [3]. Antimicrobial tolerance levels may increase through gene exchange between the two important pathogens, *Salmonella* Heidelberg and *Escherichia coli* [4].

The presence of *bla*_TEM_ genes β-lactamases related to ampicillin resistance has been reported in isolates of the family Enterobacteriaceae for more than 80.9% of the samples tested [5] and in different environments [6,7,8]. The *bla*_TEM_ gene is one of the most common variants of genes conferring beta-lactamase resistance to bacteria such as *E. coli* and *Klebsiella pneumoniae,* as well as other Gram-negative bacteria [9]. Its spread is facilitated by horizontal gene transfer (HGT), such as bacterial conjugation, a feature universally conserved among bacteria [10]. Resistance mediated by the *bla*_TEM_ gene [11] stands out for its involvement in food matrices in the dissemination and microevolution of antibiotic-resistant bacteria [12,13,14]. It presents itself as a major challenge in the control of Gram-negative bacteria [15,16].

In *S.* Typhimurium, ampicillin resistance rates exceed 75% [17]. In response to this health challenge [18], research is focused on finding actions that reinforce key interventions in combating antibiotic-resistant microorganisms and the mechanisms via which resistance genes are spread [19,20]. In addition, monitoring AMR in *E. coli* is already a mandatory indicator to verify the occurrence of bacterial phenotypes resistant to clinically important antibiotics such as carbapenems, colistin, and beta-lactams in the environment [21] and in products of animal origin in Europe [21,22].

Horizontal gene transfer is a major contributor to increased diversity in the microbial genomic repertoire [23], and conjugation is one of the most common mechanisms for transferring antibiotic resistance plasmids [24]. This process is characterized by the dynamics of direct contact and assembly of the type IV secretion system (T4SS) of a donor bacterium and DNA transfer to a recipient bacterium [25,26]. The overall rate of conjugation is determined not only by the type of plasmid that can be transferred but also by the cell physiology of the strains forming the conjugation pair, as well as the environmental conditions and the availability of energy [27], which directly influence the efficiency of conjugation and the subsequent growth of the transconjugants [28].

Although extensively investigated for different factors, approaches to inhibit bacterial conjugation are still understudied. Thus, potential targets settle on the conjugative elements and the membrane assembly system of plasmid-encoded conjugation [29]. Therefore, the approach that aims to associate anti-conjugative compounds with already demonstrated microbial properties to prevent the spread of resistance without acting as a growth inhibitor is of interest [30].

Essential oils (EOs) are known for their antimicrobial properties that, in association with nanoparticles, have advantages in overcoming limitations of use in food, such as odor, besides increasing their efficacy and stability, promoting greater solubility and bioavailability. They are also natural and sustainable and can be incorporated into the food industry. Nanoparticles can also improve the targeted delivery of EOs to specific tissues or cells [31]. Promising work on the use of essential oil-loaded nanoparticles for the control of pathogenic microorganisms is already underway [32,33], and researchers have already demonstrated that turmeric-loaded lipid nanocarriers (NLCs) can effectively control wild-type microorganisms such as *Pseudomonas aeruginosa* [34]. However, little is known about the action of nanoparticles carrying bioactive compounds in gene transfer processes and the use of new molecules that can prevent or decrease bacterial conjugation. Understanding the dynamics of HGT allows for explaining the complexity of some modulating factors of the transfer, and in vitro measurements of this process generate effective answers about the transfer rates among microorganisms.

Furthermore, the central objective of this work was to evaluate the action of chicken juice and whey coproducts on the frequency of bacterial conjugation of the *bla*_TEM_ gene and the use of sage and olibanum NLCs as inhibitors of gene transfer.

## 2. Results

### 2.1. Physicochemical and Morphological Characteristics of Lipid Nanocarriers

The association of NLCs with different solid lipid matrices confer stability for delivering the EOs of olibanum (NpO) and sage (NpS). In a previous study, the nanocomposites were evaluated for particle size, polydispersity index, zeta potential, biocompatibility, and cytotoxicity to evaluate antimicrobial activity in *Campylobacter jejuni* [35]. These characteristics were in line with the previous data and showed excellent results for size and polydispersity index. The zeta potential (mean: −36.6 to −31) indicated the stability of the nanoparticles when evaluated by three subsequent measurements at 25 °C for the NpO and NpS formulations (Table 1). 

**Figure 1:**
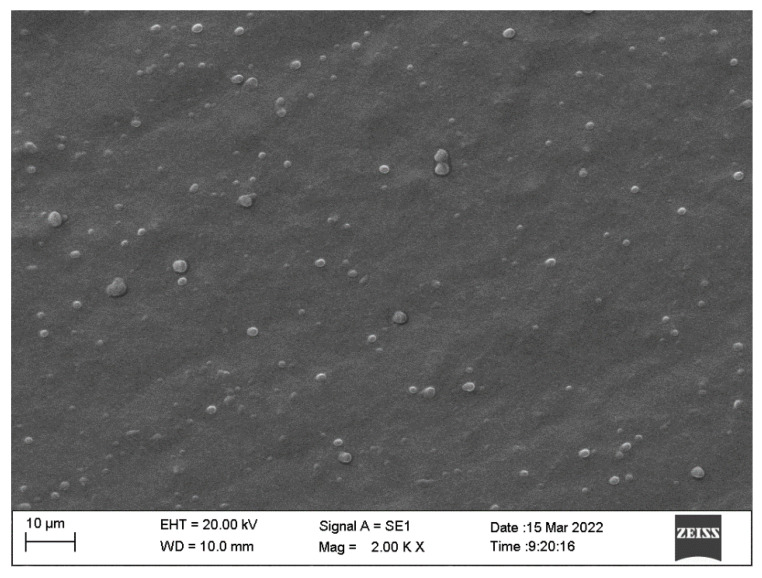
Scanning electron microscopy (SEM) image of the essential oil lipid nanocarrier formulation. Image taken at 20,000× magnification.

The average particle diameter ranged from 257.6 to 228.3 nm for sage and olibanum NLCs, respectively, with a polydispersity index equivalent to 0.15 and a zeta potential of approximately 30–35 mV. Although the size of the NLCs showed a slight increase in the presence of different EOs, the stability between the formulations remained within the parameters presented over time, indicating that incorporating the bioactive compounds does not significantly influence the size of the particles. The values obtained for the polydispersity index (PDI) were below 0.2 for all systems, without significant variations. This demonstrates that the nanocomposites were in an acceptable distribution state, with low variability and no aggregation. Furthermore, the image obtained with SEM at the 10-micrometre scale evidenced the morphology and distribution of the nanometric particles, characterizing a polydisperse system with particles of homogeneous spherical morphology, with the absence of agglomerations (Figure 1).

### 2.2. Minimum Inhibitory Concentration (MIC) of Sage and Olibanum Lipidic Nanocarriers 

The assay to evaluate the antimicrobial activity of the formulations with nanoparticles was confirmed by different in vitro experiments. The minimum bactericidal concentration (MBC) was 25 mg/mL for NpS and above 50 mg/mL for NpO, with a MIC of 1.8125 mg/mL for both carriers. From this, the optimum concentration of 0.390625 mg/mL (390.62 mg/L) was selected for NpS and NpO, considered ideal for the inhibition of bacterial conjugation but insufficient to kill the bacteria; it also did not result in a significant difference in the counts of *E. coli* and *S.* Heidelberg (*p* > 0.05) compared to the control (Table 2).

The counts of donor, recipient, and transconjugant bacteria in the absence or presence of inhibitors did not significantly differ among the replicates (*p* > 0.05). Despite evaluating the effects of different factors on overall HGT rates, few studies have encompassed and weighed selective pressures within natural environments. At the macro-level, the frequency of conjugation (FCR) was 2.23%, whereas when supplementing the conjugation process with CJ (chicken carcass co-product) and SL (homemade Mina’s cheese co-product, Federal University of Uberlândia Farm, MG/Brazil), the FCR was 6.22% and 8.16%, respectively.

### 2.3. Spectral Evaluation and Co-Product Supplementation

**Figure 2:**
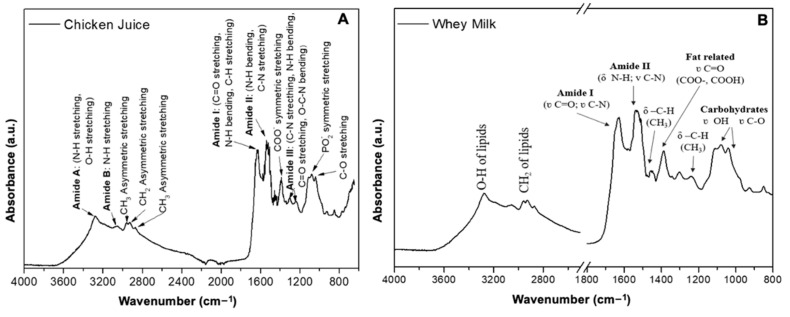
Comparison of FTIR spectra for chicken juice (**A**) and whey (**B**). a.u.: absorbance units.

One of the characterization techniques used to evaluate the different constituents of SL and CJ is infrared vibrational spectroscopy, in which bands referring to the stretching of bonds present in the molecules (constituents) are observed. The region of the vibrational spectra adopted was 4000 to 650 cm^−1^. The bands for SL, shown in Figure 2, were identified between 3400 and 3000 cm^−1^, referring to the stretching of lipid bonds ν(O-H) and ν(CH2). The fat in the whey was confirmed due to the presence of the C-H bond stretching band (3000–2800 cm^−1^) [36] from triglycerides [37].

In the wavenumber region between 1700 and 1000 cm^−1^, there are vibrational modes of the binding bands related to fatty acids, proteins, and polysaccharides, and the intensity of the bands observed can be related to the concentration of proteins present in the samples [38]. The most intense bands observed at 1700 and 1500 cm^−1^ refer to the bond stretches ν(C=O), ν(C-N) and δ(N-H), ν(C-N), respectively, from the amide functional groups (I and II) present in the peptide bonds [39]. The band observed at 1080 cm^−1^ can be attributed to stretching the O-H bond present in carbohydrates, such as lactose [40].

The bands referring to bond stretching (νN-H, νO-H, νCH2, νCH3) were observed in a region of 3500–3300 cm^−1^ for Amide A and at 3200–3000 cm^−1^ for Amide B from protein constituents of the sample chicken juice. In the region of 1800 to 1600 cm^−1^, the bands of the bonds (νC=O, δN-H, νC-H) were observed for Amide I, whereas those in the region of 1570–1470 cm^−1^ (δN-H, νC-H) were observed for Amide II and those at 1350–1250 cm^−1^ (νC-N, δN-H, νC=O, δO-C-N) for Amide III; these bands are characteristic of the protein content of the sample [41]. In the region between 1220 and 900 cm^−1^, we observed bands referring to the stretching of νC-O, νP-O2- bonds from the polysaccharides [42].

### 2.4. Gene Exchange and Inhibitors of the Conjugation Process

**Figure 3:**
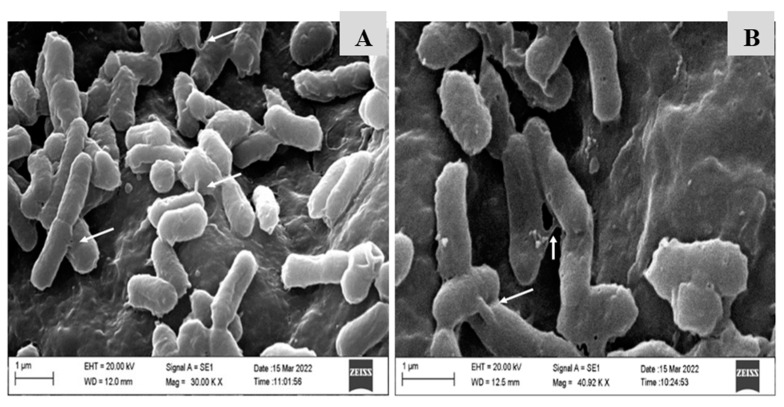
Scanning electron microscopy (SEM) image of bacterial conjugation between *Salmonella* Heidelberg and *Escherichia coli* (**A**,**B**), demonstrating multiple pili for horizontal gene transfer. Images were taken at magnifications of 30,000× and 40,000×.

Via SEM, we visualized gene transfer based on the presence of sexual pili (Figure 3). The samples presented a high cell density, enabling stabilization and contact between pairs. As shown in Figure 3A,B (arrows), the occurrence of sexual pili connections was homogeneous in all samples.

**Figure 4:**
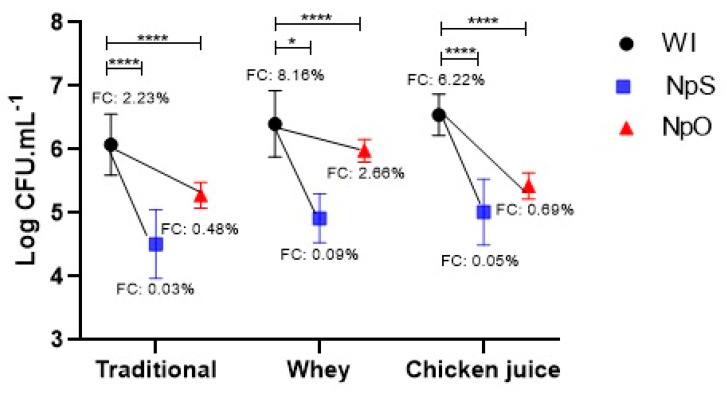
Comparison of conjugation frequencies obtained with and without 0.39 mg/mL of NLCs with essential oils of sage (NpS) and olibanum (NpO). WI: Without inhibitors; T: traditional; W: Whey; CJ: chicken juice. FCR: Receptor conjugation frequency in percent. **** *p* < 0.0001 and * *p* < 0.01, statistical difference by one-way ANOVA for the comparison with and without treatment with NLCs.

Conjugation in a liquid medium was chosen for the tests based on the conjugation rates obtained in a previous study [43]. We used the pair of microorganisms for which we determined an increase of more than 2.5 to 3.5 orders of magnitude in the percentage of frequency of conjugation in the presence of CJ and SL when compared to the traditional process (without the presence of coproducts). The pre-established 3-h period for evaluating the conjugation process represents the shift flow configurations present in animal product industries. The presence of the coproduct’s whey and chicken exudate in this interval increased the gene transfer by 0.4 and 0.5 log CFU/mL (*p* = 0.013), respectively. The use of NpS had the greatest impact, with expressive inhibition results in all treatments, and reduced the conjugation efficiency by up to 124-fold when animal-origin coproducts were added. The NpO showed more effective results in the traditional conjugation model (reduction of four orders of magnitude) and the presence of CJ (reduction of 9 orders of magnitude). Moreover, it is essential to note that SL promoted a higher increase in conjugation frequency when compared to CJ, resulting in a greater challenge for inhibition with NCL, which was reduced by three orders of magnitude (Figure 4).

### 2.5. Molecular Analyses

Molecular analyses by conventional PCR demonstrated the presence of the *bla*_TEM_ gene in all transconjugant colonies for the three compound treatments evaluated and their respective supplements. This result confirms the phenotypic characteristics of the transconjugant plates containing sodium azide (160 µg/mL) and ampicillin (90 µg/mL).

## 3. Discussion

Bacterial conjugation events occur in various environments and are ubiquitous [44,45]. They can also occur in bacterial communities present even in animal products, such as fresh milk [46] and chicken meat, and the genes can potentially be transferred during food consumption [47]. Mimicking the food production chain in this study, with the use of CJ or SL coproducts, evidenced the construction of a model in this area associated with environmental conditions that more accurately represent the extrinsic factors that can influence HGT, such as microbial mobility, fluid movements, and the microenvironment that can disturb the stabilization of the mating pair [24], depending on the junction between receiver and donor through the pilus [48].

The conjugation process, in the presence of organic matter coproducts (chicken juice or whey), demonstrated that the intrinsic factors present in the production chain, especially in production systems with continuous and automated batches, can promote the growth of certain unwanted bacterial subpopulations [49]. This makes their presence a primary factor in assessing HGT in these environments.

The gradual process of DNA transfer in a liquid medium and under agitation ensured efficient recombination rates when incubated for 3 h, equivalent to approximately one-third of a work shift in the food industry. The frequency of conjugation is generally higher in environments under agitation compared to static environments, probably due to the increased number of collisions between cells [50].

Under these conditions, the gene exchange process of the *bla*_TEM_ gene was maximized by three and four orders of magnitude when incorporating coproducts of CJ and SL, respectively. Under optimal conditions, the culture of recipient cells can be converted into transconjugants within 30 min of the addition of donor cells at an optimal temperature of 37 °C so that the mating pair requires only 5–7 min to allow DNA transfer [51]. Thus, active growth in a nutrient-rich environment and under optimal conditions would favor conjugation by sustaining the energy expenditure required for this process [52]. 

Few studies have focused on the extrinsic factors that interfere with bacterial conjugation (BC) [47], i.e., the conditions that allow microbial fitness for plasmid transport costs [53]. Environments with an optimal temperature [54] or that have nutrient availability [55] simulate natural conditions of conjugation rates under different experimental conditions [56]. However, these procedures are simplified. They are ideal for measuring specific rates and isolating modulating factors [57]. There is strong evidence that bacterial growth increases conjugation frequency, and most research on conjugation is performed on growing bacterial populations or populations subjected to growth conditions [50].

The composition of coproducts present in processed food can be evaluated by the FTIR spectroscopy technique, which demonstrates the structural differences in the molecules of different types of foods and determines the use and bioavailability of nutrients [58]. In this sense, using whey as an inducer of recombinant protein production by *Escherichia coli* improved the fitness and efficiency of protein expression without interfering with membrane fluidity and permeability [59]. Thus, the use of lactose as a carbon source, present in SL, by *E. coli* maximized the metabolism and microbial machinery due to the greater availability of micronutrients. After using glucose and in the presence of lactose, *E. coli* grows faster compared to when it uses other carbon sources as intracellular lactose is degraded by the enzyme LacZ (encoding β-galactosidase) into galactose and glucose, of which the latter can directly enter the Embden-Meyerhof-Parnas pathway [60]. The interval between 1,450 and 600 cm^−1^, present in CJ, is considered the fingerprint region, with characteristic bands coming from different biomolecules besides proteins, such as lipids, phospholipids, and nucleic acids [61]. As for SL, enteric bacteria such as *E. coli* can use nitrogen-containing organic compounds as sole nitrogen sources [62]. Recently, it has been observed that this microorganism can use DNA as an excellent source of carbon and nitrogen to grow rapidly [63]. These results show that in addition to isolating certain transfer pathways, the full biological context requires integrating biophysical knowledge with environmental and growth conditions [57].

In this context, we found that CJ and SL contained components of the amide grouping with effects on biological activity [64]. These results may facilitate a more reliable characterization of the extrinsic factors that may interfere with and maximize gene exchange and can aid in discovering the contribution of these components individually or together to the ratio and frequency of conjugation in animal product environments.

In addition, previous studies have demonstrated the importance of dairy [65] and meat products [66] as reservoirs of antibiotic resistance genes and in maintaining the transmission of antibiotic resistance, especially when the cold chain and proper heat treatment are not maintained [47,67]. In environments with low nutrient levels [56], in the food industry, coproducts offer an optimal environment for multiplication and gene exchange. In a previous study, conjugation efficiency was maximized in environments of higher hydrodynamic stress, with consequent exposure to greater encounters between donor and recipient cells, favoring conjugation compared to static environments [68]. The establishment of contact between donor and recipient cells can be considered as limiting the rate of microbial conjugation and determining plasmid specificity [10].

Naturally, the environmental niches present in food processing, from primary production to the industry [69], are critical for the persistence of resistance genes, representing an essential link for transfer to the microbiota of the final consumer and the environment [70,71]. Furthermore, plasmid transfer rates can be affected by the number of plasmids the donor cell possesses since gram-negative bacterial cells can have between one and five simultaneous pili [72] and perform multiple plasmid transfers to increase transfer and survival rates [73]. In this process, contact with the recipient cell favors the assembly of multiple T4SS systems in the donor cells, triggering the transfer of more than one copy of plasmid DNA to the recipient cell [23]. The type IV conjugative secretion system (T4SS) is a multi-subunit complex [74] anchored to the membrane and responsible for elaborating a long extracellular filament—the conjugative pilus—that is essential for ADN transfer [75].

As a measure of interfering with this process, determining the minimum inhibitory concentration of compounds that stop or decrease conjugative ADN transfer may be an effective option for controlling HGT [76]. Platforms using nanotechnology associated with biocompatible compounds, such as EOs, have gained prominence for their versatility [77]. In the present study, the optimal dilution of these nanocompounds was determined before the conjugation experiments, with low concentrations for both NLCs, ensuring that most primary applications are safe [35,78,79,80] and avoiding interference with bacterial growth and anti-conjugation activity [81]. This approach consists of associating EOs derived from medicinal plants and highlighting the action of promising new compounds that are bacterial conjugation inhibitors (COINS) in vitro. EOs are a rich source of bioactive compounds that can modify bacterial resistance [82], not only by hindering the growth of gram-negative bacteria but also by inhibiting transfer in a specific manner through interference with ADN replication [83]. In parallel, the adhesion interface and physicochemical effects play an important role in modulating the conjugative transfer (CT) process, irrespective of the microbial physiological response [84]. 

Regarding the association of these EOs with nanoparticles to inhibit conjugation, the physical effects promoted by spherical-shaped NpS present greater interactivity with the bacterial membrane, resulting in tension and stretching processes [85] in the plasma membrane, which is the primary assembly center for the initiation of the conjugation process [23]. Microspheres with adequate particle size and distribution are primary factors in optimizing the results because they influence the release rate of the bioactive compound, interfering with the results obtained [86].

Some promising conjugation inhibitors (COINS), such as unsaturated fatty acids (2-hexadecanoic acid—2HDA), seem to affect TrwD, belong to the ATPase secretion superfamily, binding to bacterial membranes [74], which hinders pilus biogenesis and ADN translocation [87]. Indeed, the current mechanisms explaining the functionality of the structural components of type VI secretion do not exhibit specificity with target receptors on the cell surface, but this can be explained by a mechanical process (symbolically called “shot and bomb”) in which perforation of the receptor cell by force or enzymatic activity of the pilus occurs [10]. Unsaturated fatty acids can prevent plasmid transfer in vitro [88], but NLCs carrying bioactive have not been described as potential COINs. Using the delivery system (NLC), we observed an expressive reduction in conjugation frequency promoted by NpS, which caused HR to decrease by 74 orders of magnitude without animal industry coproducts. However, when the challenge was greater, in the presence of SL and CJ, this inhibition reached 90 and 124 orders of magnitude, respectively. In previous studies, CJ supplementation allowed the maintenance of viable microorganisms. It maximized the results for the formation of strong biofilms, evidencing that this by-product facilitates the survival of bacterial cells in the production chain, increasing the challenges in food safety [89,90]. 

It is important to stress that the NLC complex associated with sage and olibanum EOs interfered with the conjugation process, even at low concentrations. Therefore, modern experimental approaches, such as the use of bioactive compounds associated with NLCs [33,91,92], may favor a greater interaction with cells due to a higher surface-to-mass ratio [93]. Plants are a source of bioactive compounds, and a current approach is to isolate species that allow new insights into the inhibitory mechanisms of HGT at the molecular and cellular levels [10] as a measure to control antimicrobial resistance.

Robust screenings of bioactive molecules have already shown a 100-fold reduction in plasmid transfer frequencies [83]. Among these compounds, dehydrocrepninic acid (DHCA) [94] from tropical plant seeds [95] and tanzawaic acids A and B inhibit the conjugation of IncF and IncW plasmids and IncW and IncFII conjugative plasmids, respectively, without affecting cell growth [83]. 

The essential oil of olibanum is derived from species of the genus *Boswellia* [96] and presents, as main markers, boswellic acid in commercially standardized extracts at concentrations of 37.5% to 65% [22]. It also contains two promising agents, namely p 11-keto-β-boswellic acid (KBA) and 3- O-acetyl-11-keto-β-boswellic acid (AKBA,) which are present in all *Boswellia* species and are responsible for its pharmacological effects [97]. 

Similarly, *Salvia officinalis* L. (sage) of the Lamiaceae family is rich in phenolic compounds, mainly rosmarinic acid and yunnaneic acid [98], and used in medicines, cosmetics, and food flavoring [99]. In addition to the already-known activities, it has recently been evaluated regarding the modulation of bacterial virulence and resistance, with promising results [100].

Studies investigating the long-term effects of these formulations on stability and toxicity are crucial for validating new systems [101]. These formulations showed a greater versatility of action in different pathogen control processes and lower toxicity in previous studies [35], highlighting their potential to prevent HGT in the food production chain. Thus, the use of biocompounds is focused on preventing the mobilization of plasmids in important environments from the food safety point of view.

In conclusion, two NLCs associated with sage and olibanum EOs were evaluated regarding the inhibition of the conjugation process. The animal production coproducts SL and CJ influenced HGT. As a mitigating measure, the use of NLCs associated with EOs of sage and olibanum promoted a significant reduction in FC.

## 4. Materials and Methods

### 4.1. Origin of Strains

The study used a strain of *S.* Heidelberg that showed phenotypic resistance to five antimicrobials (ampicillin, cefoxitin, sulfamethoxazole, tetracycline, and clavulanic acid) which represent four important classes in human and veterinary medicine [102]. This strain was investigated for resistance genes by conventional PCR and contained the CMY-2, *bla*_TEM_ genes and *luxS*, linked to the *quorum sensing* system. The *E. coli* strain J53AzR was used as the recipient strain [103].

### 4.2. Nanostructured Lipidic Carriers

The NLC formulations, composed of different matrices of solid lipids (shea butter and ucuuba) and liquid lipids (EOs of sage and olibanum) were previously formulated and tested on *Campylobacter jejuni* [35] at the Biotechnology Institute (Federal University of Uberlândia). Briefly, the solid and liquid lipids (EOs of salvia and olibanum at 50 mg/mL) were heated in a water bath (10 °C above the melting point of the solid lipid). Subsequently, an aqueous solution of the biosurfactant Plantaren 1200® was heated to the same temperature as the lipid phase and added to the oil phase under high-speed (10,000 rpm) stirring for 2 min in an Ultra-Turrax mixer (IKA Werke Staufen®, Staufen, Germany). Next, the systems were ultrasonicated for 12 min in a Vibracell high-end sonicator (Sonics & Mat. Inc.^®^, Danbury, CT, USA) operated at 500 W and 20 kHz, alternating 30-s cycles (on/off). Sage (NpS) and olibanum (NpO) nanoparticle formulations were stored at 25 °C. The physicochemical stability of the nanoparticles was previously validated [35] and re-evaluated in this work at three different time points to verify long-term stability.

### 4.3. Determination of the Minimum Inhibitory Concentration (MIC)

The MIC values of essential oil-associated lipid carriers for *S.* Heidelberg and *Escherichia coli* J53AzR were determined using the broth microdilution method (EUCAST, 2022). Eight different concentrations were tested: 6.25, 3.125, 1.5625, 0.78125, 0.390624, 0.1953, 0.0976, and 0.0488 mg/mL. Briefly, bacterial suspensions were prepared at a concentration corresponding to 0.5 on the McFarland scale in sterile 0.85% saline solution and then inoculated into microplates containing Müller–Hinton broth with the two compounds alone at the different concentrations, followed by incubation at 37 °C for 18–24 h. For control, we used a culture medium without the addition of bacteria and a culture medium with the addition of bacteria and without the addition of the respective compounds. 

Readings were performed visually with the determination of the MIC, corresponding to the lowest concentration where no turbidity was observed. At concentrations immediately below the MIC, a bacterial count was performed to check for a difference between the counts of the control groups compared to the formulations. For the tests, a 10-μL aliquot of each product concentration was plated on soy tryptone agar (TSA), and after growth, the colony-forming units were counted and the results expressed in log_10_, followed by a statistical evaluation to ensure that there were no differences in growth between the groups. All assays were performed in three replicates and three repetitions.

### 4.4. Chicken Juice and Whey

The addition of animal by-products, namely chicken juice (CJ) and whey (SL), was performed to mimic food industry conditions [89]. The CJ was collected from a commercial chicken carcass frozen and thawed at 25–30 °C. Subsequently, it was centrifuged three times at 14,000 rpm for 10 min at 4 °C, followed by filtration of the supernatant through a 0.22-μM pore size filter (Kasvi®) to remove contaminants. Similarly, the whey was prepared and sterilized, collected from homemade Mina’s cheese (Jersey breed animals) produced by enzymatic coagulation. Subsequently, 100 µL of the filtered by-product was inoculated into a TSA plate to verify the absence of microbial growth. Both CJ and SL were stored at −20 °C.

### 4.5. Fourier-Transform Infrared (FTIR)

The composition of the coproducts was evaluated by FTIR. A diamond disk was used as the crystal material in the ATR unit as an internal reflection element. For collection, 20 μL of CJ and SL each was applied to a high-throughput system based on aluminum wafers and heated at 60 °C on a hotplate for 3 min, followed by the application of pressure to provide contact with the crystal. Spectra were collected by scanning in the absorbance mode from 4000 to 650 cm^−1^, and 32 scans were performed per analysis at a resolution of 4 cm^−1^. The infrared spectra were recorded on a portable ATR-FTIR spectrophotometer coupled to an attenuated total reflectance unit (Agilent Technologies, Agilent Cary 630, Santa Clara, CA, USA). After this evaluation, the spectral data were processed and analyzed using OriginPro 8.0 and MS Excel. 

### 4.6. Bacterial Conjugation and Inhibition of HGT by Nanocarriers

The evaluation of the conjugation process followed the methodology proposed with adaptations [104]. Initially, the HS and CE strains were individually reactivated in the brain- and heart-infused pre-enrichment broth (BHI, Sigma-Aldrich^®^, Darmstadt, Germany) at 37 °C for at least 6 h and then repotted onto TSA (Biokar^®^, Pinhais, PR), with overnight incubation at 37 °C. After growth, approximately five colonies of each strain (donor and recipient) were selected and inoculated separately in sterile Falcon tubes containing 3 mL of Luria Bertani broth (LB-Neogen^®^, Lansing, USA/Canadá) and incubated at 37 °C in an orbital plate shaker with 60 rpm rotation for approximately three hours. After incubation, absorbance was measured (O.D._600nm_) in a microplate reader (Perlong DNM-9602) to check if the exponential phase was reached (O.D. between 0.4 and 0.5).

After absorbance determination, donor and recipient were individually subjected to serial decimal dilution (10^−1^ a 10^−8^) in 0.85% saline (Êxodo Cientifica^®^, Ortolândia, Sp). and 100 μL of each dilution was added to the surface of MacConkey agar (Biokar^®^) supplemented with the antibiotic’s ampicillin (Vetnil^®^, Louveira, Sp) at a concentration of 90 μg/mL (for *S.* Heidelberg) and sodium azide (Sigma-Aldrich^®^) at a concentration of 160 μg/mL (*E. coli* J53Az^R^) to phenotypically express their resistance. The plates were incubated at 37 °C for 48 h, with the subsequent counting of colony-forming units (CFU). The groups submitted to the conjugation assays for 3 h at 37 °C at 20 rpm were prepared in the following proportions:

(i) Traditional conjugation (no supplements): 180 μL of the donor strain (*S.* Heidelberg) and 720 μL of the recipient strain (*E. coli* J53Az^R^) represented the co-culture in a 1:4 ratio, added to 100 μL of each NLC (salvia or olibanum).
(ii) SL coproduct conjugation (5%): 170 μL of the donor strain (*S.* Heidelberg) and 680 μL of the recipient strain (*E. coli* J53 Az^R^), preserving the 1:4 ratio, added to 50 μL of SL and 100 μL of each of the NLCs in separate reactions.
(iii) CJ coproduct conjugation (5%): 170 μL of the donor strain (*S.* Heidelberg) and 680 μL of the recipient (*E. coli* J53 Az^R^), preserving the ratio of 1:4, added to 50 μL of CJ and 100 μL of each of the NLCs in separate reactions.

Similarly, conjugation was performed in the absence of inhibitors, maintaining the ratio of donor and recipient (1:4):

(i) Traditional conjugation (no supplements): 800 μL of the donor and 200 μL of the recipient.
(ii) Conjugation with SL coproduct (5%): 190 μL of the donor strain, 760 μL of the recipient, and 50 μL of SL.
(iii) CJ coproduct conjugation (5%): 190 μL of the donor strain, 760 μL of the recipient, and 50 μL of CJ. After recombination, the co-cultures were subjected to serial decimal dilution (10^−1^ to 10^−8^) in 0.85% saline, followed by surface plating on MacConkey agar (Biokar^®^) supplemented with ampicillin (90 μg/mL) and sodium azide (160 μg/mL) for the determination of trans-conjugative colonies of resistant *E. coli* J53Az^R^. All assays were performed in triplicate and three repetitions.

### 4.7. Identification of Recombinants

The transfer of resistance to β-lactams was investigated by phenotypic and genotypic methods, using conventional PCR to detect the *bla*_TEM_ gene (molecular weight 643 bp). Five trans-conjugating colonies were selected from each conjugate (traditional) and treatments (NpO and NpS), with and without supplements. The colonies were inoculated into BHI broth and incubated at 37 °C overnight, followed by bacterial ADN extraction with the Wizard Genomic ADN Purification kit (Promega). The PCR was performed in a final volume of 25 μL, using the Promega master mix kit, under the amplification conditions and with the primers listed in Table 3. Amplification was performed in a thermal cycler (Eppendorf^®^) with 30 amplification cycles: denaturation at 95 °C for 5 min, annealing at 50 °C for 45 s, extension at 72 °C for 90 s, and final extension at 72 °C for 10 min. The ADN was amplified according to the standardization conditions described in Table 3, and the amplified products were visualized via 1.5% agarose gel electrophoresis using 0.5× TBE buffer (Invitrogen) and, as a molecular weight standard, 100-bp marker (Invitrogen).

### 4.8. Gene Recombination Frequency

The conjugation frequency (CRF) was measured by calculating the ratio between the number of transconjugants (T) and the number of recipients (R) at the beginning of the conjugation process (CRF: mean T/mean R) [106]. For standardization, transconjugants were counted on plates that contained between 25 and 250 colonies [107]. Transconjugants were defined as the recipient cells (*E. coli* J53Az^R^) that received the plasmid containing the ampicillin resistance gene from a donor cell (*S.* Heidelberg) at the end of the conjugation process, counted by the average value of repeats for each treatment. Calculations were performed separately for conjugations with and without the addition of SL and CJ and for the respective inhibitor treatment groups (NpO and NpS).

### 4.9. Scanning Electron Microscopy (SEM)

#### 4.9.1. SEM—Conjugation

The ultrastructure of conjugation in all treatments was evaluated by scanning microscopy, using a modified method [89], considering the growth conditions in Luria Bertani broth (LB-Neogen^®^) and conjugation at 37 °C for 3 h/20 rpm. After conjugation, samples were fixed on 1-cm^2^ (PU) slides (Habasit Cleandrive TM, Reinach, CH) and fixed with 2.5% glutaraldehyde and 2.5% paraformaldehyde in 0.1 M PBS buffer (pH 7.4) at 4 °C overnight. Subsequently, the fixative was removed, and the samples were washed with PBS buffer. The samples were post-fixed for 2 h with 1% osmium tetroxide and washed three times with PBS buffer. The dehydrated series was applied with ethanol solutions (30, 40, 50, 60, 70, 80, and 90% and then three times at 100%) for 20 min for each step. The samples were dried in a CPD (critical drying point) system (Leica EM CPD300; Leica, Wien, Austria) using liquid carbon dioxide as a transition fluid, coated with a 20-nm gold layer (SCD 050, Baltec), and exposed on a Zeiss Supra 55 FEG SEM operating at 5 kV. Images were captured with standard ZEISS SmartSEM.

#### 4.9.2. SEM—NLC

For SEM, the samples were adhered to the stub with a glass coverslip and subsequently sprayed with gold for 120 s at 30 kV. After this, the sage (NpS) and olibanum (NpO) nanoparticles were visualized in a JEOL field scanning electron microscope (model JSM 5800LV) operating at a variable voltage from 0.3 to 30 kV with a tungsten filament, using the SemAfore 5.21 imaging system software at 40,000× and 30,000× magnification.

### 4.10. Statistical Analysis

The results were subjected to percentage descriptive analysis to calculate the conjugation frequency. The transconjugant count and the gene transfer rate were log-transformed so that the normality of the residuals could be assessed. Analysis of variance, one-way ANOVA, was performed to compare the assays of the replicates and possible differences in the results obtained. For the comparison of the two variables, the t-test was used. All analyses were performed using the GraphPad Prism 8.0 software (GraphPad Software Inc., San Diego, CA, USA).

## 5. Conclusions

Two nanostructured lipid carriers associated with sage and olibanum essential oils were evaluated to act as conjugation inhibitors. Coproducts of animal origin, namely whey and chicken juice, potentiated horizontal gene transfer. As a mitigating measure, the use of a nanocarrier associated with essential oils of sage and olibanum promoted a significant reduction in conjugation frequency. The inhibitory effect of sage nanoparticles and olibanum is a promising alternative to mitigate the microevolution of resistance gene transfer in the food industry. It motivates us to conduct further studies with these molecules to control microorganisms of public health importance.

## Figures and Tables

**Table 1:** Physicochemical stability parameters of structured lipidic nanocarriers of essential oils.

Solid/Liquid Lipid	Size (nm)	PDI	Zeta Potential (mV)
Shea butter/sage EO	257.6 ± 3.00	0.147 ± 0.01	−31.0 ± 0.1
Ucuuba butter/olibanum EO	228.3 ± 1.91	0.137 ± 0.05	−36.6 ± 0.9

Solid lipids at a concentration of 100 mg/mL and liquid lipids at a concentration of 50 mg/mL. PDI: Polydispersity Index.

**Table 2:** Mean values and standard deviation of Salmonella Heidelberg and Escherichia coli counts in the presence and absence of inhibitors at a sage and olibanum NLC concentration of 0.390625 mg/mL.

	Control	NpS	* *p*	NpO	* *p*
*Escherichia coli* (log UFC)	6.88 ± 0.09	6.87 ± 0.17	>0.99	6.71 ± 0.25	0.95
*Salmonella* Heidelberg (log UFC)	7.17 ± 0.20	6.68 ± 0.42	0.13	6.72 ± 0.25	0.23

* *p*-value. NpS: nanoparticle with sage EO and NpO: nanoparticle with olibanum EO. NLC: nanostructured lipid carriers’ formulations.

**Table 3:** Primers and conditions used to identify the bla_TEM_ gene.

Gene	Sequence 5′→3′	PCR Conditions (°C)	ADN (ng)	Reference
*bla_TEM_*	CAGCGGTAAGATCCTTGAGA ACTCCCCGTCGTGTAGATAA	95 °C—5 min; 50 °C—45 s; 72 °C 90 s; 72 °C 10 min	10	[105]

## Data Availability

Not applicable.
